# Cytokine Storm in COVID-19—Immunopathological Mechanisms, Clinical Considerations, and Therapeutic Approaches: The REPROGRAM Consortium Position Paper

**DOI:** 10.3389/fimmu.2020.01648

**Published:** 2020-07-10

**Authors:** Sonu Bhaskar, Akansha Sinha, Maciej Banach, Shikha Mittoo, Robert Weissert, Joseph S. Kass, Santhosh Rajagopal, Anupama R. Pai, Shelby Kutty

**Affiliations:** ^1^Pandemic Health System REsilience PROGRAM (REPROGRAM) Consortium, REPROGRAM Immunity Sub-committee^†^; ^2^Department of Neurology & Neurophysiology, Liverpool Hospital and South Western Sydney Local Health District, Sydney, NSW, Australia; ^3^Neurovascular Imaging Laboratory & NSW Brain Clot Bank, Ingham Institute for Applied Medical Research, The University of New South Wales, UNSW Medicine, Sydney, NSW, Australia; ^4^The University of New South Wales, UNSW Medicine, Sydney, NSW, Australia; ^5^Polish Mother's Memorial Hospital Research Institute, Lodz, Poland; ^6^Cardiovascular Research Centre, University of Zielona Góra, Zielona Gora, Poland; ^7^Department of Hypertension, Medical University of Lodz, Lodz, Poland; ^8^Department of Rheumatology, University Health Network and The University of Toronto, Toronto, ON, Canada; ^9^Department of Neurology, University of Regensburg, Regensburg, Germany; ^10^Department of Neurology, Ben Taub General Hospital and Alzheimer's Disease and Memory Disorders Center, Baylor College of Medicine, Houston, TX, United States; ^11^World Health Organisation, Country Office for India, NPSP, Madurai, India; ^12^Department of Neuromicrobiology, National Institute of Mental Health and Neurosciences, Bengaluru, India; ^13^Department of Pediatric and Congenital Cardiology, Blalock-Taussig-Thomas Heart Center, John Hopkins Hospital, Baltimore, MD, United States; ^14^Johns Hopkins Bloomberg School of Public Health, School of Medicine, John Hopkins University, Baltimore, MD, United States

**Keywords:** COVID-19, cytokine storm, immunological mechanisms, autoimmunity, neuroimmunology, immunotherapies, guidelines, critical care

## Abstract

Cytokine storm is an acute hyperinflammatory response that may be responsible for critical illness in many conditions including viral infections, cancer, sepsis, and multi-organ failure. The phenomenon has been implicated in critically ill patients infected with SARS-CoV-2, the novel coronavirus implicated in COVID-19. Critically ill COVID-19 patients experiencing cytokine storm are believed to have a worse prognosis and increased fatality rate. In SARS-CoV-2 infected patients, cytokine storm appears important to the pathogenesis of several severe manifestations of COVID-19: acute respiratory distress syndrome, thromboembolic diseases such as acute ischemic strokes caused by large vessel occlusion and myocardial infarction, encephalitis, acute kidney injury, and vasculitis (Kawasaki-like syndrome in children and renal vasculitis in adult). Understanding the pathogenesis of cytokine storm will help unravel not only risk factors for the condition but also therapeutic strategies to modulate the immune response and deliver improved outcomes in COVID-19 patients at high risk for severe disease. In this article, we present an overview of the cytokine storm and its implications in COVID-19 settings and identify potential pathways or biomarkers that could be targeted for therapy. Leveraging expert opinion, emerging evidence, and a case-based approach, this position paper provides critical insights on cytokine storm from both a prognostic and therapeutic standpoint.

## Introduction

The coronavirus disease 2019 (COVID-19) pandemic has caused a public health crisis with profound long-term socioeconomic fallout. COVID-19 results from infection with the severe acute respiratory syndrome coronavirus 2 (SARS-CoV-2) virus (1). Although the vast majority of patients experience mild to moderate symptoms, the disease remains fatal in a significant proportion of those infected (2–4). Much of the critical illness associated with SARS-CoV-2 infection is believed to be the result of a hyperinflammatory process referred to as hypercytokinemia or a “cytokine storm” (5). A full understanding of the immunopathogenesis, of cytokine storm in COVID-19 patients has the potential to guide future strategies to improve early diagnosis and implement therapeutic strategies to mitigate cytokine storm-associated morbidity and mortality risks (5, 6). This article discusses the implications of hypercytokinemia for COVID-19 patients, including the risk factors for cytokine storm, potential therapeutic strategies (6), and clinical considerations, with special emphasis on patients with cancer, autoimmune diseases, and those undergoing immunosuppressive therapies.

## COVID-19 and Cytokine Storm

### Pathophysiology

Observations from the first cohort of 41 COVID-19 patients in Wuhan, which led to the discovery of the novel SARS-CoV-2 virus, revealed a cytokine profile similar to that of secondary hemophagocytic lymphohistiocytosis (sHLH), a hyperinflammatory condition triggered by viral infection (2). Patients who were admitted to intensive care unit (ICU) had higher levels of granulocyte-macrophage colony-stimulating factor (GM-CSF), interferon gamma-induced protein 10 (IP10), monocyte chemoattractant protein-1 (MCP-1), macrophage inflammatory protein 1 alpha (MIP1A), and tumor necrosis factor alpha (TNFα) compared to those who were not admitted to ICU (2). Observations from another 150 patients in Wuhan revealed that those who died of COVID-19 complications had higher serum levels of C-reactive protein (CRP), interleukin (IL)-6 and ferritin, suggesting an underlying hyperinflammatory process (3). A combination of these markers may therefore be used as prognostic markers to determine COVID-19 severity. Another study showed that patients experiencing COVID-19-related cardiac injury with the elevated levels of troponin T (TnT) also demonstrated significantly higher CRP and procalcitonin levels (up to 3–4 times more) and experienced increased morbidity and mortality (4).

Patients who die from severe COVID-19 disease experience endothelial cell infection and an endotheliitis affecting many organs (7, 8). The SARS-CoV-2 S protein binds to angiotensin converting enzyme 2 (ACE2) to enter host cells. Most COVID-19 patients present with respiratory symptoms because ACE2 receptors are expressed in vascular endothelial cells of the lower respiratory tract (9). In severe COVID-19 cases, hypercytokinemia in the lungs leads to diffuse alveolar damage, hyaline membrane formation, thrombus formation [confirmed in small vessels at autopsy (10)], fibrin exudates, and fibrotic healing. These pathologic changes result in acute lung injury and manifest clinically as acute respiratory distress syndrome (ARDS) (11). Forty percent of COVID-19 patients experience proteinuria and haematuria, suggesting kidneys infection and injury (12). COVID-19-related kidney injury occurs because ACE2 receptors are found in the kidney in the brush border of proximal tubular cells (12). Although the kidneys of COVID-19 patients examined post-mortem reveal SARS-CoV 2 antigens in the proximal tubules, the role of cytokine storm in causing kidney injury is not yet clear (13).

ACE2 receptors are also present in cardiac tissue and in the gastrointestinal tract, arguably explaining the cardiac and gastrointestinal clinical manifestations in some COVID-19 patients. Available data suggests that those with underlying cardiovascular disease, hypertension, severe dyslipidaemia, obesity, and diabetes are at high risk for severe COVID-19 disease (14), whilst other data indicates that SARS-CoV-2 infects the heart, resulting in myocarditis and myocardial infarctions (6, 7, 15–17). Patients with underlying cardiovascular disease are at increased risk of cytokine storm (4, 18) and poor outcomes. COVID-19 patients with underlying cardiovascular disease are also at higher risk of myocardial injury [with cardiac troponin (TnT) increase], as well as both atherosclerosis-related and thromboembolic events such as stroke, plaque instability, vasculitis, and myocardial infarction (7, 15, 19). COVID-19 has also been presumably linked to central nervous system (CNS) symptoms and conditions including acute necrotizing encephalitis, myalgia, and headache among others although the pathogenesis is uncertain (20–25). Owing to the lower ACE2 expression levels in the CNS tissues, it has been hypothesized that the SARS-CoV-2 *per se* can generate little inflammation (26). Recent autopsy studies found scarce evidence of inflammation (26–30). Whether the transfer of SARS-CoV-2 to CNS tissues potentiate or exacerbate cytokine storm is a subject of ongoing debate (28, 29).

### Immunosenescence and Cytokine Storm

Elderly patients, especially older males, with comorbidities, demonstrate increased susceptibility to poor prognosis or increased risk of severe condition or even fatality from COVID-19 (31). Aging is associated with a decline in immune function or “immunosenescence” (32–36). With age, the immune system can present with a series of changes, characterized by immunosenescence markers (34–36), a decrease in the generation of CD3+ T cells, an inversion of the CD4 to CD8 (CD4/CD8) T cells ratio due to the loss of CD8+ T cells (35) (increased CD4/CD8 ratio), an increase in regulatory T cells (Treg) and a decrease in B lymphocytes (34). It is postulated that COVID-19 induced cytokine storm may be contributing to the poor outcomes in elderly patients due to immunosenescence. T lymphocytes can be potentially infected by the virus (37), reducing their number due to their apoptosis. It is currently not known whether the infection of the lymphocytes themselves potentiate cytokine storm or otherwise. In a recent study employing immunomodulatory therapeutic strategy, intravenous transplantation of mesenchymal stem cells (MSCs) was effective, especially in critically severe cases, in a series of 7 patients with COVID-19 pneumonia (38). Immunomodulatory therapies targeting cytokine storm show potential for such approaches in improving outcomes and reducing mortality due to COVID-19 in elderly patients (5, 39). Future studies are required to further evaluate the efficacy of immunomodulatory therapies in preventing cytokine storm induced severe illness in COVID-19 patients in general, and elderly patients in particular (38).

### Significance of Cytokine Storm

Hypercytokinemia is an unregulated hyperinflammatory response that results from the systemic spread of a localized inflammatory response to viral or bacterial infection. Elevated cytokine levels result in endothelial dysfunction, vascular damage, and paracrine/metabolic dysregulation, thereby damaging multiple organ systems. Levels of acute-response cytokines (TNF and IL-1β) and chemotactic cytokines (IL-8 and MCP-1) rise early in hypercytokinemia, facilitating a sustained increase in IL-6. IL-6 binds to either membrane bound IL-6 receptor (mIL-6R) or soluble IL-6 receptor (sIL-6R), forming a complex that acts on gp130, regulates levels of IL-6, MCP-1 and GM-CSF via the Janus kinase-signal transducer and activator of transcription (JAK-STAT) pathway, and thereby perpetuates the inflammatory processes (39). IL-6, along with other pleiotropic cytokines, drives an acute phase response that elevates serum ferritin, complement, CRP, and pro-coagulant factors, many of them measurable through commercially available blood tests. The acute phase response of cytokine storm is relatively over-exaggerated. Since high serum levels of cytokines are inversely related to the total lymphocyte count, low levels of cytotoxic T cells may contribute to reduced viral clearance (40). Blocking upstream events related to or at the level of cytokine response, such as JAK-STAT signaling of macrophages to reduce IL-1 and IL-6 production, offers a potential therapeutic target for the cytokine storm. Cell-based target strategies may also be considered, but the time to therapeutic effect of anti-B lymphocytes directed therapies such as rituximab may be too long to be clinically relevant. Therefore, targeting the upstream events may be relatively more effective.

**Figure 1:**
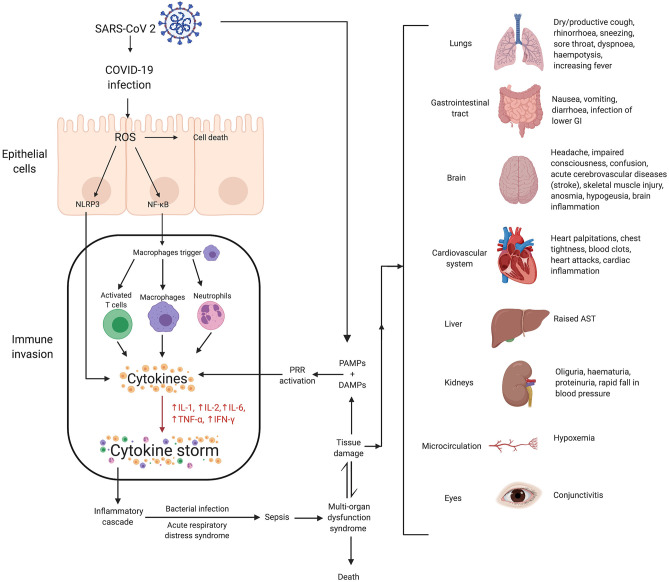
Mechanisms of SARS-CoV-2 associated cytokine storm and associated damages. Infection with SARS-CoV 2 can stimulate a hyperinflammatory immune response wherein epithelial-cell-mediated production of reactive oxygen species (ROS) can cause cell death. ROS can also stimulate the synthesis of NLRP3 and NF-κB which contribute to increased cytokine levels, and thus, the cytokine storm. This essentially causes immune invasion which can lead to clinically relevant conditions such as ARDS, sepsis, MODS and potentially even death. The organs affected as a result of MODS, and their associated symptoms, have been shown. Lower gastrointestinal (GI) is rich in ACE2 receptors and hence at higher risk of infection due to COVID-19. Twenty percent of COVID-19 patients have diarrhea as symptoms. SARS-CoV-2, severe acute respiratory syndrome coronavirus 2; COVID-19, coronavirus disease 2019; ROS, reactive oxygen species; NLRP3, (NOD)-like receptor protein 3 inflammasome; NF-κB, nuclear factor kappa-light-chain-enhancer of activated B cells; IL, interleukin; TNF, tumor necrosis factor; IFN, interferon; PAMPs, pathogen-associated molecular patterns; DAMPs, damage-associated molecular patterns; PRR, pattern recognition receptors; AST, aspartate aminotransferase; MODS, multiple organ dysfunction syndrome.

In reaction to SARS-CoV-2 infection, macrophages (41) and dendritic cells trigger an initial immune response, including lymphocytosis and cytokine release. However, the inflammatory response results in the destruction of lymphocytes attempting to stop SARS-CoV-2 infection. Lymphopenia ensues, especially in patients severely affected enough to require ICU admission (42). Cytokine production becomes rapidly dysregulated, damaging healthy cells typically first in the lungs but potentially spreading to other organs including the kidneys, heart, blood vessels, and brain. The cascade of cytokine storm-associated damage begins with disruption of the epithelial barrier in the lungs. Activation of NOD, LRR-, and pyrin domain-containing protein 3 (NLRP-3) inflammasome and the relative blunted response of histone deacetylase 2 on nuclear factor kappa betta (NFκB) complex has been suggested to be associated with cytokine storms. The epithelial barrier disruption exposes the lungs or other tissues to bacterial infection. Pathophysiological mechanisms associated with COVID-19 induced cytokine storms are shown in Figure 1 (11, 43–50).

**Figure 2:**
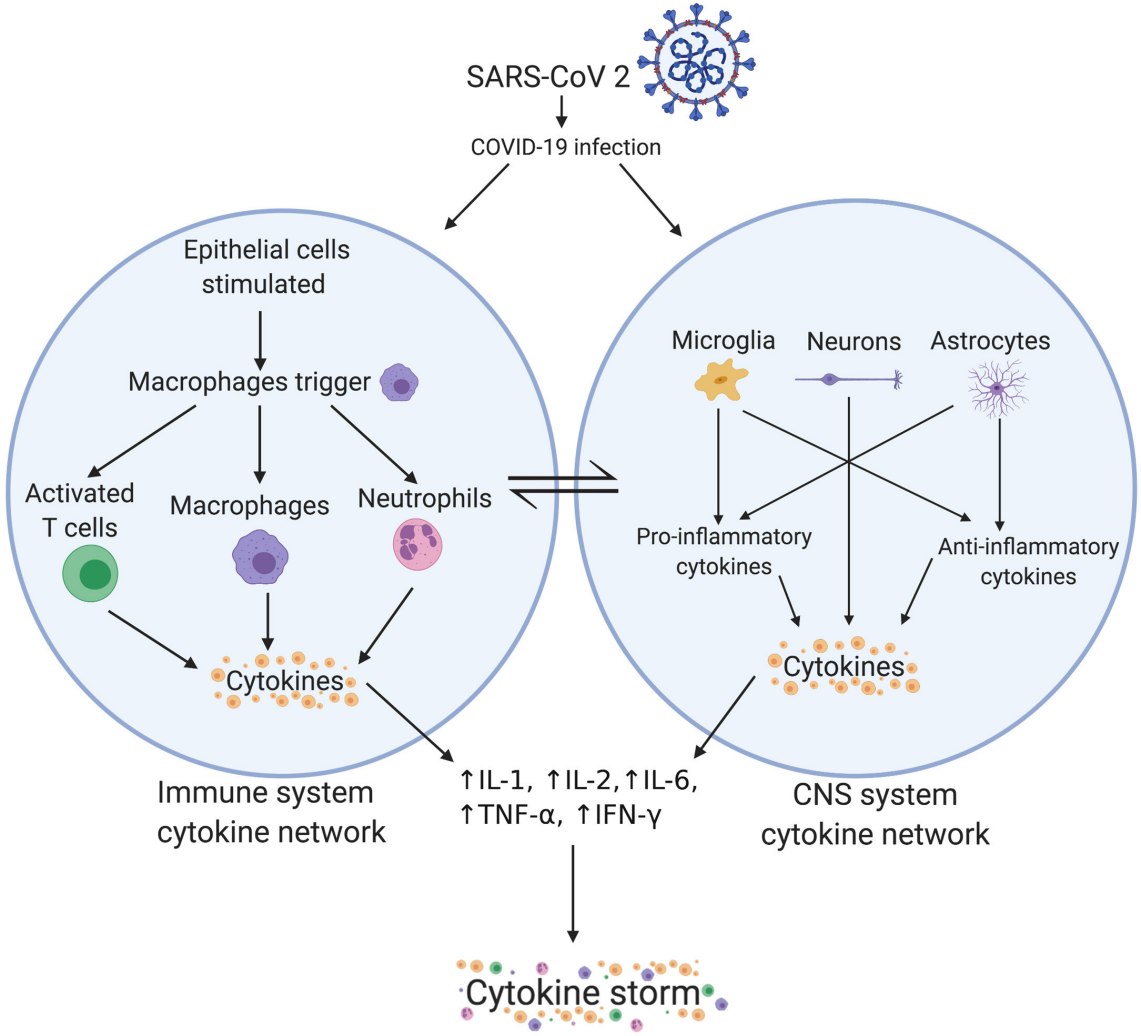
Crosstalk between immune system and CNS system cytokine networks. There is a supposed link between the immune system cytokine network and the CNS system cytokine network. Peripheral cytokines can cross the blood brain barrier to enter the CNS. Alternatively, microglia and astrocytes can also produce cytokines. Potential involvement of neurons in regulation of cytokines for example brain-derived neurotrophic factor (BDNF) and interleukin-6 levels is also plausible (51). SARS-CoV-2, severe acute respiratory syndrome coronavirus 2; COVID-19, coronavirus disease 2019; CNS, central nervous system; IL, interleukin; TNF, tumor necrosis factor; IFN, interferon.

We propose that the immune system cytokine network may also communicate with the central nervous system (CNS) cytokine network, especially when the blood-brain-barrier (BBB) is compromised. Microglia and IL-1 activation can cause increased reactive oxygen species (ROS) production, phagocytosis, apoptosis, and increased cytokine expression (see Figure 2) within the CNS (43), leading to neural tissue damage through neuroinflammation, increased oxidative stress and excitotoxicity, and dysfunction in synaptic pruning. The systemic immune system cytokine network and the CNS cytokine network influence each other through the neuropeptidergic pathway involving neurokinin C and B, neuroendocrine peptides (NPY)/gastrin-releasing peptide (GRP), SPA-GRP {SPA: [(D-Arg, D-Trp, Leu)Substance P], a derivative of substance P}, and vasoactive intestinal polypeptide (VIP). Activation of macrophages and phagocytosis, chemotaxis with neutrophils and degranulation of mast cells, and activation and proliferation of T-cells activate this pathway. Inflammatory cytokines are also be transported through the blood, which could further amplify the cytokine storm (11). We postulate that the overlapping immune and CNS cytokine networks may drive “immune hijack.” In light of these mechanisms and potentially devastating impact of COVID-19 on “high-risk” patients, specific clinical considerations for medical conditions have been discussed in the following section.

## Cytokine Storm and High-Risk Patients—Clinical Considerations for Specific Medical Conditions

Some of the severe complications associated with COVID-19 are acute respiratory distress syndrome (ARDS) (prevalence of 17–29%), acute cardiac injury including myocarditis, myocardial infarction and cardiac arrest, sepsis, multi-organ failure, ischemic stroke, Kawasaki-like syndrome in children, acute pulmonary embolism, sHLH, and secondary infections such as bacterial pneumonia (Supplementary Table 1) (3). Therefore, special considerations must guide management of patients at high risk of severe COVID-19 disease and cytokine storm, including patients with underlying coronary artery disease, obesity, cancer, primary immunoglobulin deficiencies, autoimmune conditions, as well as those receiving immunosuppressive therapies. A risk-based strategy to identify high risk patients is presented in Table 1. Measuring the viral load at different time points as well as the immune response may help optimize treatment strategies. Next, we will discuss the radiological findings consistent with hyperinflammation or cytokine storm in COVID-19 cases.

**Table 1:** Patient at “risk” of severe outcomes after COVID-19 associated cytokine storm.

**High-risk patients**	**Risk category in COVID-19 settings**	**Therapeutic strategy**
Hemodynamic instability or cardiogenic shock	Very high risk	Invasive STEMI^*^ pathway
Cardiac arrest/life-threatening arrhythmia	Very high risk	Invasive STEMI pathway
Acute heart failure	Very high risk	Invasive STEMI pathway
Recurrent intermittent ST elevation	Very high risk	Invasive STEMI pathway
Mechanical complications of myocardial infarction	Very high risk	Invasive STEMI pathway
Established diagnosis of NSTEMI based on cardiac troponins AND at least one of the following (52): 1. recurrent symptoms 2. dynamic ST/T changes (silent or symptomatic)	Very high risk	Testing followed by invasive STEMI strategy
Acute stroke	Very high risk	Acute stroke pathway
Acute meningitis/encephalitis	Very high risk	Respiratory care and ongoing monitoring; increased intracranial pressure pathway; epileptic seizure monitoring
Age ≥ 75	High risk	Respiratory care and ongoing monitoring
Solid organ or stem cell transplant patients	High risk	Respiratory care and ongoing monitoring
HIV patients	High risk	Respiratory care and ongoing monitoring
Inherited immune conditions	High risk	Respiratory care and ongoing monitoring
On immunomodulatory therapy	High risk	Respiratory care and ongoing monitoring
Undergoing cancer treatment	High risk	Respiratory care and ongoing monitoring
Obesity	High risk	Respiratory care and ongoing monitoring
Diabetes	High risk	Respiratory care and ongoing monitoring
Established diagnosis of NSTEMI based on cardiac troponins AND at least one of the following (52); 1. Diabetes mellitus or renal insufficiency (estimated glomerular filtration rate < 60 mL/min/1.73 m^2^); 2. Left ventricular ejection fraction (LVEF) < 40% or congestive heart failure; 3. Early post infarction angina or prior PCI/CABG	High risk	Non-invasive testing using CCTA, respiratory care and ongoing monitoring
Epileptic seizures, status epilepticus	High risk	Respiratory care and ongoing monitoring; seizure/status epilepticus treatment
Coronary artery disease (CAD)	Intermediate risk	Respiratory care and ongoing monitoring
Cerebrovascular disease	Intermediate risk	Respiratory care and ongoing monitoring
Cardiovascular disease (CVD)^**^	Intermediate risk	Respiratory care and ongoing monitoring
Pre-existing Hypertension	Intermediate risk	Respiratory care and ongoing monitoring
Smoking	Intermediate risk	Respiratory care and ongoing monitoring
Pneumonia	Intermediate risk	Respiratory care and ongoing monitoring

* *NSTEMI, non-ST segment elevated MI; MI, myocardial infarction; CVD, cardiovascular disease; CAD, coronary artery disease; PCI, percutaneous coronary intervention; CABG, coronary artery bypass grafting; CCTA, cardiac computed tomography angiography*.

** *CVD is linked to inflammation and oxidative stress, and patients who are not adherent to the anti-inflammatory therapy (Angiotensin-converting-enzyme inhibitors (ACEIs) or angiotensin-II-receptor-antagonists (ARBs) and statins), viruses such as SARS-CoV2 might immediately cause degranulation of macrophages and monocytes in the damaged endothelium, causing atheroma plaque instability, and increased coagulopathy*.

### Cytokine Storm and Radiological Findings

Although the association between cytokine storm and the radiological manifestations of COVID-19 pneumonia infection require further investigation, computer assisted tomography (CT), the lungs of patient with COVID-19 pneumonia typically demonstrate findings typical of underlying hyperinflammatory pathway (53, 54). On CT chest, the lungs typically demonstrate ground-glass opacities (subpleural, peripheral and bilateral) (53), bronchovascular thickening within lesions, smooth or irregular interlobular or septal thickening, air space consolidation, traction bronchiectasis, ill-defined margins, air bronchograms, and thickening of the adjacent pleura (54). IL-1β induces the production of bronchoalveolar lavage fluid, creating the ground-glass appearance (40). CT findings evolve over time (54–60). Normal CT scans may be seen in the first 3–4 days. During the intermediate stage, septal thickening and increased ground glass opacities appear (57). During the advanced stage, which is usually at 9–13 days of the disease, the features seen in the intermediate stage consolidate. After 14 days of the disease, during the resolution stage, fibrous stripes appear and typically resolve after 1 month (58–60).

### COVID-19 Associated Coagulopathy and Its Complications

Patients with COVID-19, especially younger patients, are at a higher risk of hypercoagulability and thereby experience higher rates of arterial and venous thromboses (61–64). A case series reported large vessel ischemic stroke in young asymptomatic or mildly symptomatic COVID-19 patients (62). Critically ill patients appear to experience high rates of acute venous thromboembolism (VTE) (63, 64). In 54 consecutively admitted ICU patients treated with prophylactic low molecular weight heparin since admission, 22.2% experienced VTE [predominantly deep vein thrombosis (DVT)] (63). In a retrospective study of severe COVID-19 patients admitted to ICU (*n* = 81), 25% of the patients not receiving pharmacologic VTE prophylaxis developed lower extremity DVT, and 40% of those patients died. A D-dimer level of >1.5μg/mL predicted VTE with high sensitivity and specificity (64). Another study of 191 patients reported significantly higher mortality in patients with D-dimer >1.0 μg/mL compared to those whose level was <1.0 μg/mL (65). A 31% cumulative incidence of thrombosis (from ischemic stroke, DVT, acute pulmonary embolism, myocardial infarction, systemic arterial embolism) has been reported, with pulmonary embolism being the most common thrombotic complication (81%) (66). A prothrombin time >3.0 s and a prolonged aPTT >5 s have also been reported to independently predict thrombotic complications (66).

An autopsy series microscopically confirmed the presence of platelets and thrombi in small vessels, thrombi in small vessels in the peripheral aspect of lungs, and scattered areas of diffuse alveolar damage (67). Gross pathological examination revealed small firm clots in sections of peripheral parenchyma of the lungs. Other autopsy series have revealed microthrombi in small pulmonary arterioles and diffuse alveolar damage in the majority of cases. In light of the findings of high-frequency pulmonary micro thrombosis on histology, the hypothesis of COVID-19 induced coagulopathy or hypercoagulation merits further discussion. In a large retrospective analysis of consecutive severe cases (*n* = 449), elevated D-dimer and prothrombin time were correlated with a higher mortality rate (68). However, neither aPTT nor platelet count was significantly different between mildly and severely affected patients. Elevated levels of D-dimer level may indicate secondary fibrinolysis, contributing to clinically severe manifestations of COVID-19 infections. It is noteworthy that anticoagulation significantly reduced mortality in patients with the International Society for Thrombosis (ISTH) sepsis-induced coagulopathy score of ≥4 (40.0 vs. 64.2%) (68). However, there are variations in the incidence of VTE in ICU patients across several centers. A meta-analysis of 9 studies demonstrated that D-dimer level were elevated and coagulopathy more prevalent in patient with severe disease as compared to those with mild disease (69).

The American Society of Haematology recommends VTE prophylaxis with LMWH or fondaparinux (alternative to unfractionated heparin to reduce exposure) in all hospitalized COVID-19 patients unless the risk of bleeding outweighs thrombosis risk (70). Fondaparinux can also be used in patients with a history of heparin-induced thrombocytopenia (HIT), as those patients are at 5-fold increased risk of severe COVID-19. Mechanical thromboprophylaxis should be used when anticoagulation is either contraindicated or unavailable. According to the recent guidelines of the ISTH, all patients with an elevated D-dimer (typically a 3 to 4-fold increase) should be admitted to the hospital. Fibrinogen levels should be monitored at the later stages of the disease (day 10–14) with >2.0 g/L in both bleeding and non-bleeding patients indicating disseminated intravascular coagulation. The guidelines recommend consideration of LMWH in all patients requiring hospital admission for COVID-19 except those in whom anticoagulation is contraindicated (71). Contraindications for anticoagulation with LMWH are platelet count <25 × 109/L, active bleeding, or severe renal impairment (71). Notably, either an abnormal PT nor aPTT was not listed as a contraindication for anticoagulation with LMWH. Further studies of the association of elevated D-dimer and other coagulopathy markers with cytokine storm and severity of COVID-19 clinical manifestations are warranted. Optimal anticoagulation strategies aimed at correcting or preventing coagulopathy should also be expeditiously studied (14, 18, 19).

### Immunosuppressed/Cancer Patients

Certain cancer patients, especially those with hematopoietic or lymphoid malignancies, are at higher risk of severe COVID-19 disease because they are immunocompromised (72, 73). Global Radiation Oncology has made specific recommendations about treating several types of cancers during the COVID-19 pandemic (72). Although patients undergoing chemotherapy or radiotherapy are temporarily immunocompromised, colony stimulating factors can be administered to strengthen their immune system (73). Generally, oncologists are accustomed to managing infections. However, for cancer patients infected with COVID-19, benefit to risk ratio-based chemotherapy is followed in the absence of guidelines and prospective Phase 2 evidence (74). It is recommended that cancer-related treatment be delayed if treatment provides only modest benefit and the biology of the cancer allows for delay. If radiation is being administered for palliative purposes, all alternatives including maximizing analgesics and bisphosphonates should be explored. In situations like painful bone metastases, radiation cannot be avoided (73). In such scenarios, a single 8 Gy fraction should be used because it is as effective as multiple fraction courses (74).

Hematopoietic stem cell transplantation recipients should practice self-isolation prior to transplantation. If such a patient becomes infected with SARS-CoV-2, the procedure should be delayed (75). Complete immunological recovery following stem cell transplantation may take 3 to 6 months, so self-isolation after the procedure is necessary as well. Everyone who comes in direct contact with either a stem cell or an organ transplant recipient should be vaccinated for common respiratory viruses (76). A cancer patient presenting with symptoms suggestive of COVID-19 should also be evaluated for mimics. Pneumonitis from radiation therapy for example, can be treated with corticosteroids, but this same treatment may cause pulmonary injury in COVID-19 patients (75).

HIV-infected patients should be provided sufficient supply of medications to avoid treatment gaps and to allow them to maintain a viral load below the level of detectability. HIV-infected patients who display symptoms of COVID-19 should be prioritized for diagnostic testing because they are at risk for severe complications (77).

### Autoimmune Conditions

Patients with systemic autoimmune conditions, including systemic lupus erythematosus (SLE), vasculitis, multiple sclerosis, progressive systemic sclerosis, or rheumatic disease affecting the lungs are at a greater risk of developing complications secondary to respiratory viruses (78). This increased susceptibility to lung disease may be the result of either the underlying disease or immunosuppressive treatments (79). Patients with active autoimmune conditions should continue immunosuppressive treatment because the risk of relapse is more detrimental than the risk of SARS-CoV-2 infection. Stable patients should be maintained on their current therapeutic regimen. Therapy should be changed in stable patients only if they are at a higher risk for exposure to COVID-19 or if they become infected with the disease. In this situation treatment should gradually be reduced and halted consistent with the guidelines from the American College of Rheumatology (80) and the German Society of Rheumatology (81). Corticosteroid injections into joints or soft tissues should only be reserved for severe cases (78, 82). Because psychological stress can induce flare-ups in patients with rheumatic diseases, patients experiencing anxiety, depression, or suicidal thoughts should be referred for mental health support. This can be done through telemedicine to reduce risk of COVID-19 transmission. Patients should be encouraged to maintain their daily routine, such as sleeping a consistent amount of time and maintaining a healthy diet, within the isolation of their homes (78).

## Therapeutic Strategies to Target Cytokine Storm

**Figure 3:**
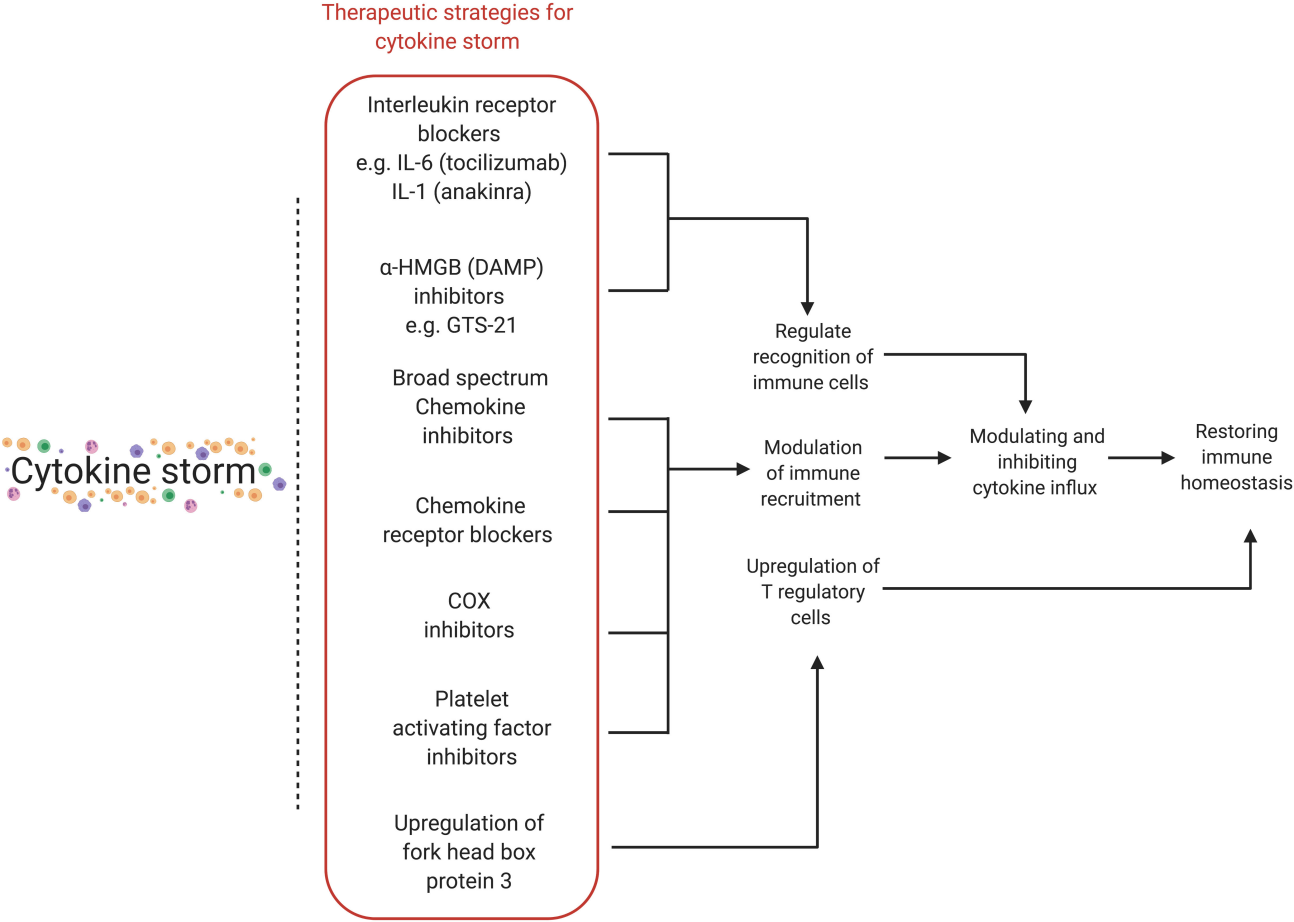
Various therapeutic strategies for targeting cytokine storm. Different stages of the hyperinflammatory immune response can be targeted for therapeutic purposes, with the final aim of modulating and inhibiting cytokine influx in order to restore immune homeostasis. HMGB, high-mobility group protein 1; DAMP, damage-associated molecular pattern; COX, cyclooxygenase.

Various stages of the cytokine storm pathway can be targeted for therapeutic effects (Table 2 and Figure 3) (110). Cytokine storm has an inciting trigger (viral infection), as well as factors potentiating pathogenic effects and perpetuating the cycle of hyperinflammation. Immunomodulation may improve outcomes even without antiviral drugs (11). A list of ongoing clinical trials targeting cytokine storm and hyperinflammation is presented (Supplementary Table 2). The patient's immune and comorbidity profile may modulate response to therapy. Drug interactions with medications used for SARS-CoV2 therapy as well as strategies targeting cytokine storm with antivirals, antiretrovirals, antimalarials (e.g., chloroquine and hydroxychloroquine), or immunomodulators (e.g., tocilizumab) may be considered on a case-by- case basis.

**Table 2:** Various immunomodulatory strategies targeting cytokine storm in COVID-19 patients.

**Strategies or agents**	**Studies and indications**	Safety/drug to drug interactions^*^	**References**
Cyclooxygenase (COX) inhibitors	The use of COX inhibitors in COVID-19 has not been evaluated. It should be used on a case-by-case basis.	Should not be used in patients with previous history of stroke, or prior heart bypass surgery (coronary artery bypass graft, or CABG). Cox-2 inhibitors (Celecoxib) have less gastrointestinal side effects than non-steroidal anti-inflammatory drugs (NSAIDS). Similar cardiovascular event risk profiles of Cox-2 and non-selective NSAIDS (ibrufen, diclofenac and naproxen). Cox inhibitors increases risk of cardio-thrombotic events, congestive heart failure.	(83)
Corticosteroids	The use of high-dose corticosteroids is not recommended in cases of COVID-19.	Mild to intermediate dose may be considered to reduce inflammation in initial treatment of cytokine storm and in specific cases of COVID-19-induced pneumonia.	(83)
Anti-tumor necrosis factor (TNFa) therapy	Anti-TNFa is widely used for several autoimmune diseases. Its use in COVID-19 should be explored.	May be protective against SARS-CoV-2 pneumonia.	(84)
Intravenous immunoglobulin (IVIg) therapy	Due to its lack of side effects, IVIg may be beneficial in COVID-19 patients especially in settings of cytokine storm or hyperinflammatory state and septic shock.	Could be explored as an alternative to corticosteroids. Low dose IVIg may require complement activation; whereas, high doses of IVIg may act directly on immune cells (85).	(86)
Angiotensin-converting-enzyme inhibitors (ACEIs) or angiotensin-II-receptor-antagonists (ARBs)	The use of ACEI/ARB is associated with lower mortality in COVID-19 in-patients.	ARBs preferable in preventing kidney failure in patients with established (diabetic) nephropathy (87). ACEIs advantageous in prevention of new onset albuminuria ACEIs have relatively higher mortality benefit than ARBs in the setting of prior MI, coronary artery disease or heart failure. ACEIs (as a monotherapy or with a diuretic) have greater benefit in recurrent stroke prevention. ARBs can be considered in patients who do not tolerate ACEIs owing to cough and angioedema.	(88)
Peroxisome proliferator-activated receptor (PPAR) agonists	PPAR agonists increase the production of anti-inflammatory cytokines and thus, may be beneficial in COVID-19 patients. PPAR-γ agonists are often used in treatment of type 2 diabetes.	PPAR-γ agonist thiazolidinediones (TZDs), like pioglitazone and rosiglitazone, have anti-inflammatory properties with potential for corrective effects on severe viral pneumonia. Nutritional ligands of PPAR-γ, such as lemongrass, pomegranate, and curcuma may be used in conjunction with PPAR pharmacological agents (89).	(90)
5′ adenosine monophosphate-activated protein kinase (AMPK) activators	AMPK activators such as metformin have direct anti-inflammatory effects. Could have benefit in reducing cytokine storm in COVID-19 infected patients.	AMPK activators has shown to increase survival rates in influenza infected animal models. Combination with pioglitazone could have added survival benefits (91).	(92)
Macrolide	The antiviral effects of macrolide may benefit COVID-19 patients.	Macrolide may reduce inflammation in infected patients.	(93)
Arbidol	Arbidol is an antiviral that has been shown to prevent COVID-19 infection.	Studies on animal model of influenza has shown benefits in reducing mortality, inflammation and lung lesion formation (94). Arbidol could be explored in targeting cytokine storm.	(95)
OX40 (CD134)	There are several limitations in the therapeutic use of OX40.	OX40–immunoglobulin fusion protein treatment has previously demonstrated clinical benefit in influenza animal models by eliminating weight loss and cachexia without preventing virus clearance (96).	(97)
Antioxidants	Antioxidants, such as vitamin C, have anti-inflammatory effects when administered intravenously.	Could be used in combination with other anti-inflammatory agents to target cytokine storm.	(98)
Suppressor of cytokine signaling (SOCS)	SOCS is involved in regulating antiviral immunity.	Could be protective against severe cytokine storm during severe COVID-19 infection.	(99)
Extracorporeal therapy	Extracorporeal therapy is a proposed mechanism to remove cytokines in septic patients	Extracorporeal cytokine removal may have protective effects on vascular integrity and could reverse cytokine storm (100).	(101)
Pyrrolidinedithiocarbamate (PDTC) ammonium	Inhibits IκB phosphorylation and thus blocks NF-κB translocation to the nucleus and reduces the expression of downstream cytokines.	PDTC ammonium may have a role in limiting cytokine storm by inhibiting reactive oxygen species (ROS) production (102).	(103)
Diacerein	An inhibitor of IL-1B—an acute response cytokine which appears in hypercytokinemia.	Diacerein attenuates inflammation in severe sepsis, and hence improves survival (104). Could be beneficial in reducing sepsis induced insulin resistance as an alternative to insulin therapy in severe sepsis cases where intensive insulin therapy is associated with adverse outcomes. Diacerein could be beneficial in managing diabetes patients infected with SARS-CoV-2.	(105)
Tranilast	An anti-allergic drug which inhibits NOD-, LRR- and pyrin domain-containing protein 3 (NLRP3) which plays an important role in the pathogenesis of COVID-19.	Tranilast has been shown to attenuate ischemia reperfusion injury by inhibiting inflammatory cytokine production and PPAR expression (106).	(107)
Statin	*In-silico* evidence on efficacy of statins as SARS-CoV-2 Mpro inhibitors	As drugs of choice—Rosuvastatin (with preference for starting a low dose and titrating up) and Fluvastatin should be administered.	(16, 108)
Chloroquine/Hydroxychloroquine	Strong anti-inflammatory activity may be of use in targeting cytokine storm.	Not recommended currently by Food and Drug Administration (FDA) and Europeans Medicine Agency (EMA) outside of the hospital setting or a clinical trial due to risk of heart rhythm problems and fatal conditions including congestive heart failure.	(109)

* *With standard therapy used for SARS-CoV-2 infection: antiviral drugs (remdesivir), antiretroviral drugs (lopinavir/ritonavir), macrolides (mainly azithromycin), anti-malaria (chloroquine and hydroxychloroquine) and anti-rheumatoid (tocilizumab)*.

### Corticosteroids and NSAIDs

Corticosteroids and NSAIDs can effectively suppress hyperinflammatory responses; however, delayed viral clearance could lead to further complications and also increase the risk of transmission. Although corticosteroids could be used acutely to target cytokine storm, their use in respiratory viral infection is associated with increased mortality, increased risk of secondary bacterial or fungal infections, and prolonged ICU admission. Furthermore, corticosteroids may mask COVID-19 related fever. As such, corticosteroids and NSAIDs are not recommended for routine management of COVID-19 patients (40), despite a theoretical benefit in reducing cytokine storm risk.

### Targeting Interleukins

**Figure 4:**
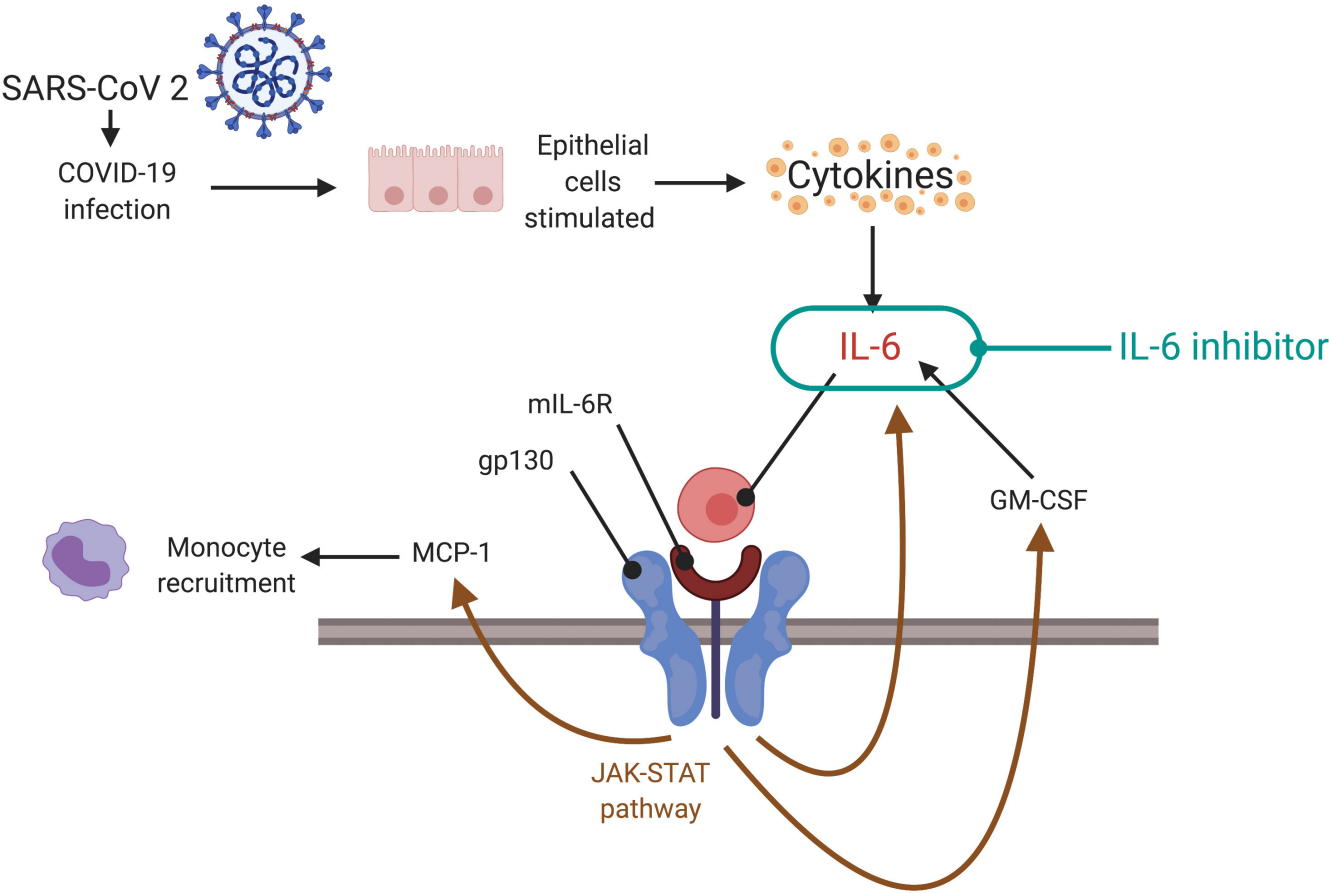
Targeting cytokine storm via the JAK-STAT pathway. During a cytokine storm, there are increased levels of IL-6 which can form a complex with mIL-6R to act on gp130. Gp130 regulates levels of IL-6, MCP-1, and GM-CSF via the JAK-STAT pathway. This could facilitate the cytokine storm. Inhibition of the JAK-STAT pathway, potentially using IL-6 inhibitors or direct inhibition of signaling, can be a therapeutic strategy (depending on the timing—indicated preferably at later stages of illness, not in early phase, or at clinical signs of cytokine storm). SARS-CoV-2, severe acute respiratory syndrome coronavirus 2; COVID-19, coronavirus disease 2019; IL, interleukin; mIL-6R, membrane bound interleukin-6 receptor; gp 130, glycoprotein 130; MCP-1, monocytes chemoattractant protein-1; GM-CSF, granulocyte-macrophage colony-stimulating factor; JAK-STAT, janus kinase/signal transducer and activator of transcription.

Interleukin-6 (IL-6) plays a key role in cytokine storm (102). Blocking IL-6 is another potential therapeutic strategy (Figure 4) (44). Tocilizumab is a monoclonal antibody against IL-6 receptor (IL-6-R) that binds to membrane-bound and soluble IL-6-Rs (mIL-6R and sIL-6R), thus preventing the downstream signal transduction of IL-6 on binding to membrane protein gp130 (102). A study of 21 tocilizumab-treated COVID-19 patients revealed that clinical manifestations improved following administration (111). Tocilizumab is undergoing phase IV clinical trials (ChiCTR2000029765) and has been approved for use in treating COVID-19 pneumonia and raised IL-6 levels in China. The Italian Regulatory Drug Agency is undertaking phase II trials of tocilizumab in COVID-19 patients (TOCIVID-19) (44). The pro-inflammatory effects of IL-6 occur via the trans-signaling pathway using sIL-6R. On the other hand, the anti-inflammatory and regenerative effects of IL-6 involve the cis-signaling pathway via the mIL-6R, present on macrophages, neutrophils, some T lymphocytes and hepatocytes. Tocilizumab is not selective for the sIL-6R and may inhibit mIL-6R, thereby causing negative side effects such as upper respiratory tract infections. Recombinant soluble gp130 protein (sgp130) may be an alternative to tocilizumab because it binds to sIL-6R, thereby reducing its pro-inflammatory effects when it binds to IL-6 (112). IL-1 inhibitors may be an alternative for treating COVID-19 hypercytokinemia. A phase III clinical trial of anakinra showed survival benefit without increased adverse effects (40). IL-37 and IL-38 could be evaluated as therapeutic options for COVID-19 because they inhibit the pro-inflammatory effects of IL-1 (113).

### Janus Kinase Inhibitors

SARS-CoV-2 enters host cells via receptor-mediated endocytosis, which is regulated by numb-associated kinases (NKA) such as adaptor complex protein 2 (AP2)-associated protein kinase (AAK1) and G-associated kinase (GAK). The high affinity AAK1 blocker ruxolitinib is under investigation for treating COVID-19 (ChiCTR2000029580). To achieve NAK inhibition, toxic doses of AAK1 blockers are required. Baricitinib can inhibit both AAK1 and GAK (approved dosage of 2–4 mg daily) and can selectively inhibit JAK 1 and 2, thus reducing the inflammatory effects of Il-6 via the JAK-STAT signaling pathway. Furthermore, baricitinib can be considered in combination antiviral and anti-inflammatory therapies due to its minimal interaction with cytochrome P450 (CYP) enzymes and low plasma protein binding (40, 114). Early reports show promise of baricitinib combined with antiviral therapy in COVID-19 patients (115).

## Decision Making Based on Cytokine Storm

**Figure 5:**
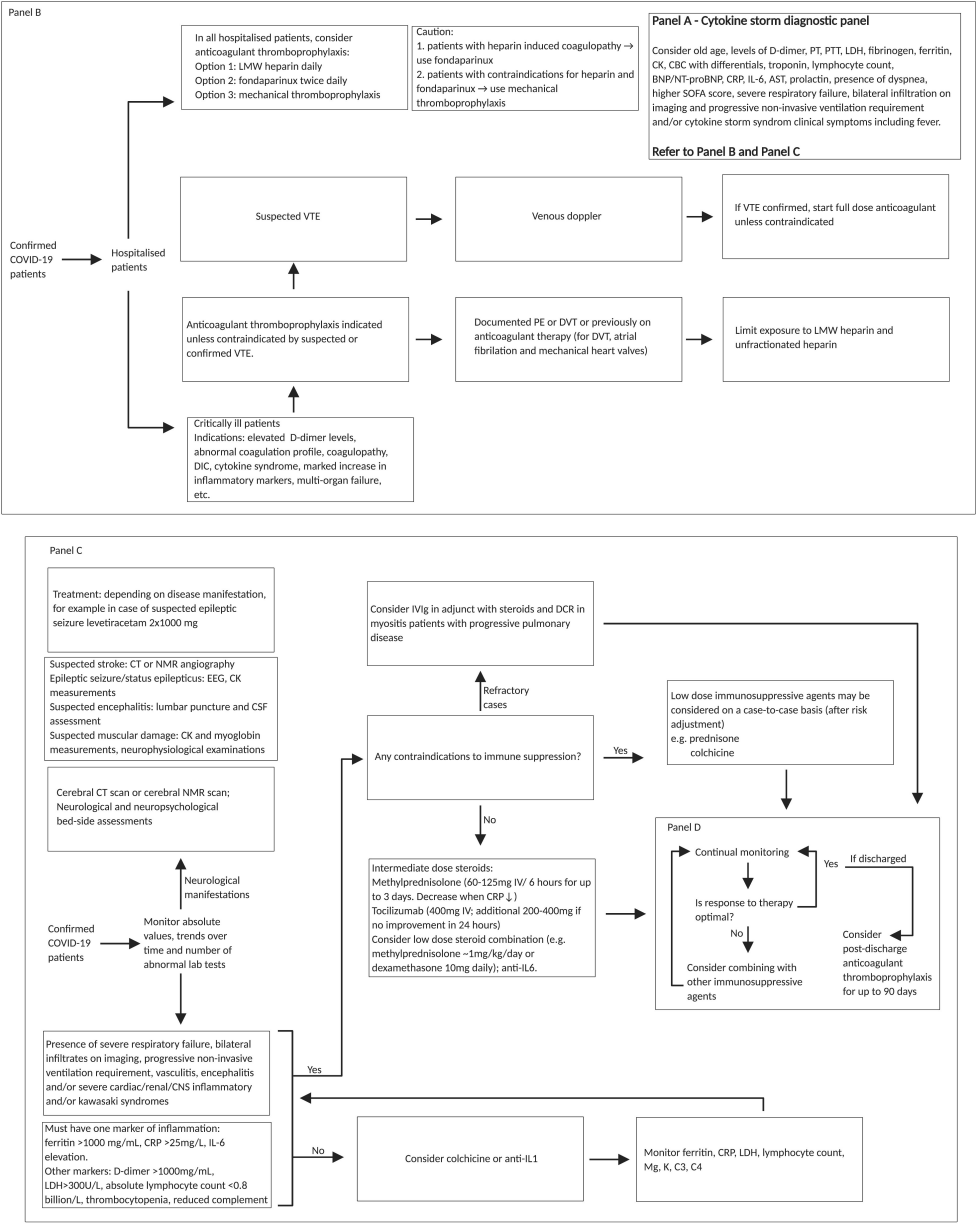
REPROGRAM consortium pathway for targeting cytokine storm in severe or critically ill COVID-19 patients. Diagnostic panel for risk factor assessment of cytokine storm associated prognosis of COVID-19 patients could include (Panel A: on top right) (99): older age, dyspnoea, higher SOFA score, IL-6, lymphocyte count; cardiac troponin; BNP/NT-proBNP (if clinical suspicion of heart failure); one marker of inflammation (Ferritin > 1,000 mg/mL, CRP > 25 mg/L, and Il-6 elevation); presence of severe respiratory failure, bilateral infiltration on imaging and progressive non-invasive ventilation requirement, D-dimer > 1,000 mg/mL; LDH > 300 U/L; absolute lymphocyte count < 0.8 billion/L; PCT level (>0.5 ng/mL), and AST > 40 U/liter (61, 116–119). In low-resourced settings, cytokine release syndrome clinical symptoms could be used in the absence or limited availability of diagnostic panels. Fondaparinux is a synthetic pentasaccharide factor Xa inhibitor. Fondaparinux binds antithrombin and accelerates its inhibition of factor Xa. It is chemically related to low molecular weight heparins. Patients with CNS involvement should have cerebral CT or MRI scan and in the if a stroke is suspected also a CT angiography or MRI angiography, in case of epileptic seizures or status epilepticus an EEG and in case of suspected encephalitis a lumbar puncture for cerebro-spinal fluid assessment. Also, bedside neuropsychological assessments are of value. In addition, assessment of CK and myoglobin are of value (neurophysiology as well, but this is not so important acutely). Treatments should include: antiepileptics (for example, levetiracetam 2x1000 mg) and depending on disease condition. SARS-CoV-2, severe acute respiratory syndrome coronavirus 2; COVID-19, coronavirus disease 2019; IV, intravenous; PT, prothrombin time; PTT, partial thromboplastic time; LDH, lactate dehydrogenase; CK, creatine kinase; CBC, complete blood count; BNP, brain natriuretic peptide; NT-proBNP, N-terminal pro hormone brain natriuretic peptide; CRP, c-reactive protein; IL, interleukin; AST, aspartate aminotransferase; SOFA, sequential organ failure assessment score; LMW, low molecular weight; VTE, venous thromboembolism; PE, pulmonary embolism; DVT, deep vein thrombosis; DIC, disseminated intravascular coagulation; CT, computed tomography; NMR, nuclear magnetic resonance; EEG, electroencephalogram; CSF, cerebrospinal fluid; IVIg, intravenous immunoglobulin; DCR, direct current cardioversion; CNS, central nervous system; Mg, magnesium; K, potassium; C, complement component; PCT, procalcitonin.

Because of the association between cytokine storm and severe COVID-19 complications, we propose a cytokine storm-based diagnosis and management workflow for patients with or suspected of COVID-19 (Figure 5 and Table 1) (120). Our proposal expands on the multidisciplinary evidence-based guidelines currently used in the diagnosis and treatment of cytokine storm linked macrophage activation syndrome and sHLH (121). The Surviving Sepsis Campaign COVID-19 panel recommends the use of moderate-dose steroids for intubated patients with ARDS (10 mg dexamethasone daily, or 60 mg/day methylprednisolone) (122).

## Conclusion and Discussions

The diagnosis and management of cytokine storm are clinically challenging and controversial due to lack of proven treatment. Our proposed algorithm may be used as a possible approach (Figure 5) (120–122); however clinical decision should be based on individual patient profile and disease severity. Immunosuppressive agents such as steroids or immunomodulating drugs such as anti-IL6 monoclonal antibodies like tocilizumab, are relatively high priced, unavailable in low resource setting, and may be in short supply during the COVID-19 pandemic even in developed countries. The neuroinvasive potential of COVID-19 (24, 25) and the association between neuroinvasion and cytokine storm need further consideration (123). We recommend longitudinal follow-up of COVID-19 patients with and without the cytokine storm to understand the specific immunopathological mechanisms and biomarkers for severe disease. After cytokine storm resolves, an immunologic memory of the SARS-CoV-2 infection will likely persist (124), raising the possibility of either relapse or reinfection in previously COVID-19 positive patients who subsequently cleared the infection. Limiting damage from a hyperimmune response during both the acute phase and the cytokine storm is a target for further research. There could be a decoupling mechanism of cytokines that may attenuate the cytokine storm and preserve memory (110). Understanding the various pathophysiological mechanisms linked to cytokine storm could be used to develop targeted diagnostic and therapeutic strategies for critically ill COVID-19 patients.

## Data Availability Statement

The original contributions presented in the study are included in the article/Supplementary Material, further inquiries can be directed to the corresponding author/s.

## Author Contributions

SB devised the project, the main conceptual ideas, including the workflow targeting cytokine storm, proof outline, and coordinated the writing and editing of the manuscript. SB and AS wrote the first draft of the manuscript. SB encouraged AS to investigate and supervised the findings of this work. All authors discussed the results and recommendations and contributed to the final manuscript. The opinions expressed in this article are those of the authors and do not necessarily represent the decisions, official policy, or opinions of the affiliated institutions.

## Conflict of Interest

The authors declare that the research was conducted in the absence of any commercial or financial relationships that could be construed as a potential conflict of interest.
