# Supplementary material for: Cytokine Storm in COVID-19—Immunopathological Mechanisms, Clinical Considerations, and Therapeutic Approaches: The REPROGRAM Consortium Position Paper
Source: Front Immunol. 2020 Jul 10;11:1648. doi: 10.3389/fimmu.2020.01648 (PMC7365905; doi:10.3389/fimmu.2020.01648)
Supplement: Supplementary file 1 [file Table_1.DOCX]

**SUPPLEMENTARY INFORMATION**

**Title: Cytokine Storm in COVID-19 - Immunopathological Mechanisms, Clinical Considerations and Therapeutic Approaches: The REPROGRAM Consortium Position Paper**

**Short Title:** Cytokine Storm in COVID-19 era - The REPROGRAM^†^ recommendations

**Authors:** Sonu Bhaskar, Akansha Sinha, Maciej Banach, Shikha Mittoo, Robert Weissert, Joseph S. Kass, Santhosh Rajagopal, Anupama R Pai, Shelby Kutty

**Supplementary Table 1.** Cytokine storm in pathological conditions.

**Supplementary Table 2.** Ongoing studies targeting cytokine storm and/or hyperinflammation in COVID-19

**Supplementary Table 1. Cytokine storm in pathological conditions.**

| **Disease/Condition** | **References** |
| --- | --- |
| Graft-versus-host disease | ^1^ |
| Multiple organ dysfunction syndrome (MODS)^⤊^ | ^2^ |
| Acute pancreatitis | ^3^ |
| Severe gut graft-versus-host disease | ^4^ |
| SARS | ^5^ |
| Respiratory syncytial virus vaccine-enhanced disease | ^6^ |
| Lewis lung carcinoma and B16 melanoma cells | ^7^ |
| Tuberculosis and HIV-co-infected patients | ^8^ |
| Avian influenza | ^9^ |
| Pediatric oncology patient | ^10^ |
| 2009 Influenza A virus infection | ^11^ |
| Rickettsioses Patients | ^12^ |
| Sepsis | ^13,14,15^ |
| Acute Coronary Syndromes: | ^16^ |
| Adiposity | ^17^ |
| Influenza-associated encephalopathy | ^18^ |
| COVID-19 | ^19-21^ |
| Hemophagocytic lymphohistiocytosis | ^22-24^ |
| Child with chronic granuloma disease | ^25^ |
| Ebola’s virus | ^26^ |
| Juvenile Arthritis | ^27^ |
| Mastocytosis | ^28^ |
| Capillary leak syndrome | ^29^ |
| Obesity | ^30^ |
| Erdheim-Chester Disease | ^31^ |
| Infection with dengue virus (DENV) | ^32^ |
| Oxiplatin associated side effect in metastatic colon cancer | ^33^ |

^⤊^subsequent to surgical insult such as a major surgery, severe trauma, extensive burn, and systemic sepsis

**Supplementary Table 2.** Ongoing studies targeting cytokine storm and/or hyperinflammation in COVID-19

| **Drug/diagnostic test/device under investigation** | **Phase of trial** | **Reference**  **(Clinical Trial Identifier)** |
| --- | --- | --- |
| Drug: ruxolitinib | Expanded access | NCT04355793 |
|  | 3 | NCT04362137 |
|  | Not applicable | NCT04361903* |
|  | 2 | NCT04359290 |
|  | 2 | NCT04338958 |
|  | 2\|3 | NCT04348071 |
| Drug: anakinra | 3 | NCT04362111 |
| Drug: methylprednisolone | 2 | NCT04355247 |
| Drug: etoposide | 2 | NCT04356690 |
| Drug: hyperbaric oxygen | 2\|3 | NCT04327505 |
| Drug: clazakizumab | 2 | NCT04343989 |
| Drug: tocilizumab and methylprednisolone | 3 | NCT04345445 |
| Drug: tocilizumab | 2 | NCT04335071 |
|  | 2 | NCT04315480 |
|  | Not applicable | NCT04306705* |
|  | 2 | NCT04331795 |
| Drug: traditional Chinese medicine prescription | 3 | NCT04323332 |
| Drug: placebo, thalidomide | 2 | NCT04273581 |
| Diagnostic test: standard 12-lead ECG, NT-proBNP, echocardiography | Not applicable | NCT04355884* |
| Drug: Levamisole Pill + Budesonide+Formoterol inhaler\|, Lopinavir/Ritonavir + hydoxychloroquine | 2\|3 | NCT04331470 |
| Drug: Colchicine | 3 | NCT04350320 |
| Drug: Drug Isotretinoin | 3 | NCT04353180 |
| Drug: Pyridostigmine Bromide | 2\|3 | NCT04343963 |
| Drug: RoActemra | 2 | NCT04322773 |
| Drug: hydroxychloroquine | 3 | NCT04325893 |
|  | 3 | NCT04360759 |
| Drug: cholecalciferol | 3 | NCT04344041 |
| Drug: Recombinant human plasma gelsolin | 2 | NCT04358406 |
| Drug: Dexmedetomidine Injectable Product | Not applicable | NCT04358627* |
| Drug: Dornase Alfa Inhalation Solution [Pulmozyme] | 2 | NCT04359654 |
|  | 3 | NCT04355364 |
| Drug: Siltuximab  Drug: Methylprednisolone | 2 | NCT04329650 |
| Drug: Baricitinib | 2\|3 | NCT04340232 |
| Non-interventional | Non-applicable | NCT04355624* |
|  | Non-applicable | NCT04347460* |
| Other: Discontinuation of ARB/ACEI\|Other: Continuation of ARB/ACEI | Not applicable | NCT04338009 |
| Device: control group | 1 | NCT04353674 |
| Device: cytokine absorption | Not applicable | NCT04361526 |
| Device: vv-ECMO + cytokine adsorption | Not applicable | NCT04324528 |
| Device: SLEDD with a L-MOD | 1 | NCT04353674 |
| Dietary supplement: oral nutrition supplement | 4 | NCT04323228 |
| Dietary supplement: ketogenic diet | Not applicable | NCT04358835 |
| Biological: anti-SARS-CoV2 convalescent plasma | Early phase 1 | NCT04345679 |
| Biological: Autologous Adipose MSC's | 1 | NCT04352803 |
| Biological: Emapalumab, Biological: Anakinra | 2\|3 | NCT04324021 |

*****= trials which are observational. All other trials are interventional.

**Supplementary References**

1. Ferrara, J. L., et al. (1993). "Cytokine storm of graft-versus-host disease: a critical effector role for interleukin-1." Transplant Proc **25**(1 Pt 2): 1216-1217.
2. Aikawa, N. (1996). "[Cytokine storm in the pathogenesis of multiple organ dysfunction syndrome associated with surgical insults]." Nihon Geka Gakkai Zasshi **97**(9): 771-777.
3. Makhija, R. and A. N. Kingsnorth (2002). "Cytokine storm in acute pancreatitis." J Hepatobiliary Pancreat Surg **9**(4): 401-410.
4. Fowler, D. H., et al. (2004). "Clinical "cytokine storm" as revealed by monocyte intracellular flow cytometry: correlation of tumor necrosis factor alpha with severe gut graft-versus-host disease." Clin Gastroenterol Hepatol **2**(3): 237-245.
5. Huang, K. J., et al. (2005). "An interferon-gamma-related cytokine storm in SARS patients." J Med Virol **75**(2): 185-194.
6. Boukhvalova, M. S., et al. (2006). "The TLR4 agonist, monophosphoryl lipid A, attenuates the cytokine storm associated with respiratory syncytial virus vaccine-enhanced disease." Vaccine **24**(23): 5027-5035.
7. Kushida, S., et al. (2006). "Artificial cytokine storm combined with hyperthermia induces significant anti-tumor effect in mice inoculated with lewis lung carcinoma and B16 melanoma cells." Int J Hyperthermia **22**(8): 699-712.
8. Ruhwald, M. and P. Ravn (2007). "Immune reconstitution syndrome in tuberculosis and HIV-co-infected patients: Th1 explosion or cytokine storm?" AIDS **21**(7): 882-884.
9. Us, D. (2008). "[Cytokine storm in avian influenza]." Mikrobiyol Bul **42**(2): 365-380.
10. Parsons, M. (2010). "Cytokine storm in the pediatric oncology patient." J Pediatr Oncol Nurs **27**(5): 253-258.
11. Cheng, X. W., et al. (2011). "Three fatal cases of pandemic 2009 influenza A virus infection in Shenzhen are associated with cytokine storm." Respir Physiol Neurobiol **175**(1): 185-187.
12. Bhavnani, S. K., et al. (2013). "How Cytokines Co-occur across Rickettsioses Patients: From Bipartite Visual Analytics to Mechanistic Inferences of a Cytokine Storm." AMIA Jt Summits Transl Sci Proc **2013**: 15-19.
13. Wen, H., et al. (2010). "Plexin-A4-semaphorin 3A signaling is required for Toll-like receptor- and sepsis-induced cytokine storm." J Exp Med **207**(13): 2943-2957.
14. Chousterman, B. G., et al. (2017). "Cytokine storm and sepsis disease pathogenesis." Semin Immunopathol **39**(5): 517-528.
15. Yao, Y., et al. (2017). "alpha-Lactose Improves the Survival of Septic Mice by Blockade of TIM-3 Signaling to Prevent NKT Cell Apoptosis and Attenuate Cytokine Storm." Shock **47**(3): 337-345.
16. Cirillo, P., et al. (2014). "Local cytokine production in patients with Acute Coronary Syndromes: a look into the eye of the perfect (cytokine) storm." Int J Cardiol **176**(1): 227-229.
17. Mirsoian, A., et al. (2014). "Adiposity induces lethal cytokine storm after systemic administration of stimulatory immunotherapy regimens in aged mice." J Exp Med **211**(12): 2373-2383.
18. Nara, A., et al. (2015). "An unusual autopsy case of cytokine storm-derived influenza-associated encephalopathy without typical histopathological findings: autopsy case report." Am J Forensic Med Pathol **36**(1): 3-5.
19. Mehta, P., et al. (2020). "COVID-19: consider cytokine storm syndromes and immunosuppression." Lancet **395**(10229): 1033-1034.
20. Wu, D. and X. O. Yang (2020). "TH17 responses in cytokine storm of COVID-19: An emerging target of JAK2 inhibitor Fedratinib." J Microbiol Immunol Infect.
21. Muema, D. M., et al. (2020). "Association between the cytokine storm, immune cell dynamics, and viral replicative capacity in hyperacute HIV infection." BMC Med **18**(1): 81.
22. Wei, A., et al. (2020). "Hemophagocytic lymphohistiocytosis resulting from a cytokine storm triggered by septicemia in a child with chronic granuloma disease: a case report and literature review." BMC Pediatr **20**(1): 100.
23. Parsi, M. and K. Dargan (2020). "Hemophagocytic Lymphohistiocytosis Induced Cytokine Storm Secondary to Human Immunodeficiency Virus Associated Miliary Tuberculosis." Cureus **12**(1): e6589.
24. Halyabar, O., et al. (2019). "Calm in the midst of cytokine storm: a collaborative approach to the diagnosis and treatment of hemophagocytic lymphohistiocytosis and macrophage activation syndrome." Pediatr Rheumatol Online J **17**(1): 7.
25. Wei, A., et al. (2020). "Hemophagocytic lymphohistiocytosis resulting from a cytokine storm triggered by septicemia in a child with chronic granuloma disease: a case report and literature review." BMC Pediatr **20**(1): 100.
26. Kennedy, J. R. (2020). "Phosphatidylserine's role in Ebola's inflammatory cytokine storm and hemorrhagic consumptive coagulopathy and the therapeutic potential of annexin V." Med Hypotheses **135**: 109462.
27. Mahajan, S., et al. (2020). "Diacylglycerol Kinase zeta Regulates Macrophage Responses in Juvenile Arthritis and Cytokine Storm Syndrome Mouse Models." J Immunol **204**(1): 137-146.
28. Valent, P. (2020). "KIT D816V and the cytokine storm in mastocytosis: production and role of interleukin-6." Haematologica **105**(1): 5-6.
29. Siddall, E. and J. Radhakrishnan (2019). "Capillary leak syndrome: a cytokine and catecholamine storm?" Kidney Int **95**(5): 1009-1011.
30. Ramos Muniz, M. G., et al. (2018). "Obesity Exacerbates the Cytokine Storm Elicited by Francisella tularensis Infection of Females and Is Associated with Increased Mortality." Biomed Res Int **2018**: 3412732.
31. Christophi, G. P., et al. (2017). "Erdheim-Chester Disease presenting with histiocytic colitis and cytokine storm." J Gastrointestin Liver Dis **26**(2): 183-187.
32. Kuczera, D., et al. (2018). "Highlights for Dengue Immunopathogenesis: Antibody-Dependent Enhancement, Cytokine Storm, and Beyond." J Interferon Cytokine Res **38**(2): 69-80.
33. Dembla V. Idiosyncratic Reaction with Cytokine Storm Associated with Oxaliplatin. *Cureus*. 2016;8(5):e615. Published 2016 May 19. doi:10.7759/cureus.615
